# The Promotion of Technology Acceptance and Work Engagement in Industry 4.0: From Personal Resources to Information and Training

**DOI:** 10.3390/ijerph17072438

**Published:** 2020-04-03

**Authors:** Monica Molino, Claudio G. Cortese, Chiara Ghislieri

**Affiliations:** Department of Psychology, University of Turin, 10124 Turin, Italy; monica.molino@unito.it (M.M.); chiara.ghislieri@unito.it (C.G.)

**Keywords:** industry 4.0, technology acceptance, work engagement, personal resources, training

## Abstract

Thanks to the rapid advances of technology, we are currently experiencing the fourth industrial revolution, which is introducing several changes in how organizations operate and how people learn and do their work. Many questions arise within this framework about how these transformations may affect workers’ wellbeing, and the Work and Organizational Psychology is called upon to address these open issues. This study aims to investigate personal and organizational antecedents (resilience, goal orientation and opportunities for information and training) and one consequence (work engagement) of technology acceptance within factories, comparing white- and blue-collar workers. The study involved a sample of 598 workers (white-collar = 220, blue-collar = 378) employed at an Italian company who filled in a self-report questionnaire. In both samples, the multi-group structural equation model showed a positive relationship between resilience, opportunities for information and training, and technology acceptance, which in turn showed a positive association with work engagement. All indirect effects were significant. This study investigated the motivational dynamics related to the introduction of new technologies within factories involving the little-studied population of blue-collar workers. Results highlighted the importance of providing information and opportunities for training to all employees, in order to support Industry 4.0 transformations without impacting on workers’ motivation.

## 1. Introduction

The fourth industrial revolution, also called Industry 4.0, especially in Europe, is already underway. Rapid advances in technology, digitalization, smart technologies, automation and the industrial internet have enabled and characterized its progression. As with any previous industrial revolution, this one, too, has important economic, political and social implications, and deeply modifies how organizations function and how people work [1]. According to Schwab [2], the fourth industrial revolution is also different from the previous ones, since (1) it evolves at an exponential, rather than linear, speed; (2) it disrupts almost all industries; and (3) it is able to impact production, management, and governance.

In general, Industry 4.0 refers to a series of transformations related to the use of several new technologies that will lead to more flexible, automated and interconnected manufacturing processes. What defines this revolution is the presence of Cyber Physical Systems (CPSs), the combination of enabling technologies which can facilitate the interconnection between different subjects, hardware, software and humans [3]. More specifically, CPSs achieve three fundamental functions: generating and obtaining data; proceeding with their computation and aggregation; and supporting the final decision. Among technologies typical of Industry 4.0, the most important are the Internet of Things, big data, advanced robotics, and additive manufacturing. 

Many benefits of Industry 4.0 may be acknowledged [4]. First, economic benefits are made possible by reducing warehouse and maintenance costs, quality costs and consumption of raw materials and energy. Second, quality is improved by a higher customization of products and customer satisfaction, and by advanced and more stable processes. Moreover, wider data availability supports predictions and decision-making and accelerates all processes. Some benefits can be found also for workers, such as improved safety and environmental protection, more autonomy and independent decision-making, and the development of new skills [5,6].

On the other side, some risks emerge. New technology implementation often fails because of employee resistance [7] and because of the inadequate management of its effects on other organizational elements [8]. Workers’ opposition may arise because of distrust, the feeling of being controlled and fear of job loss [5,9,10]. Moreover, the lack of technical and digital skills and the need for adequate training is one of the most significant obstacles among those reported in the literature, far more critical compared to the need for investments, IT security, company size, changes in the cultural or business model, and the lack of adequate infrastructure [4]. While communication and information programs are crucial to raise awareness about changes in the whole company, training plays an important role for those employees who have to use the new technology. Particularly, training allows them to learn how to use the technology and to develop positive attitudes and perceptions about the technology [11].

In light of this premise, the aim of this study was to analyse technology acceptance among white- and blue-collar workers employed in an Italian manufacturing company that was introducing innovative systems. More specifically, the study intended to analyse the antecedents of technology acceptance, considering personal (resilience and goal orientation) and organizational (opportunities for information and training) dimensions, and their relationship with work engagement. In doing so, the study highlighted the role of organizations in the promotion of public health. Work engagement, indeed, which is a state of motivation, enthusiasm and energy caused by work, is considered one of the work-related wellbeing dimensions [12] and, more specifically, has been associated to psychological and physical health at work [13]. Enhancing positive work experiences and promoting employees’ work engagement is an organization’s responsibility towards public health, which becomes even more important in the current Industry 4.0 era, where new technologies are changing the relationship between individuals and their work, with potential costs for their wellbeing [1].

### 1.1. Technology Acceptance

Technology acceptance literature is aimed at studying how individuals’ perceptions affect their intentions to use technology as well as its actual usage. Understanding the dynamics that can influence people’s acceptance of new technologies has become an increasingly central issue given the massive spread of new tools and devices. Several authors have tried to explain technology acceptance and many models are available in the psychological and sociological literature today [14]. 

The Technology Acceptance Model (TAM) [15] is the one that has received the most attention. The TAM is formulated from the Theory of Reasoned Action [16] and postulates that an individual’s perceptions about a new type of information technology (IT) predicts his/her intention to use the technology, and intention implies actual usage [15]. According to the TAM, intention to use technology depends on two beliefs: “perceived usefulness, defined as the extent to which a person believes that using an IT will enhance his or her job performance and perceived ease of use, defined as the degree to which a person believes that using an IT will be free of effort” [17] (p. 275). Moreover, the model posits that the effect of all external variables, such as design features, on intention to use is mediated by these two key beliefs, in other words, perceived usefulness and perceived ease of use.

A third belief construct has been proposed for the TAM by Mathieson and colleagues [18]. They introduced perceived resources and defined them as “the extent to which an individual believes that he or she has the personal and organizational resources needed to use an information system” (p. 89). The perceived resources construct contributes to the understanding of technology acceptance because it focuses on the perceptions of the environment where the technology is being implemented and the characteristics of the technology itself. Perceptions of barriers to use and perceptions of organizational support would, respectively, have a positive and negative effect on one’s intention to use a new technology. Several empirical and longitudinal studies demonstrated that the TAM is able to explain about 40 percent of variance in intention to use a technology and the use itself [17]. 

Another prominent theory in the study and application of technology acceptance is the Unified Theory of Acceptance and Use of Technology (UTAUT) [19], which was proposed as a comprehensive synthesis of previous technology acceptance models and theories, including TAM and the Theory of Planned Behaviour [20]. According to UTAUT, new technology acceptance and/or usage behaviour itself are influenced by four constructs [19]: performance expectancy (the belief that using the technology will help the person in their job performance), effort expectancy (the degree of ease in using the technology), social influence (the individual’s perception that he or she should use the new technology according to important others), and facilitating conditions (the degree to which a person perceives the presence of organizational supports for the use of the technology). In 2012, Venkatesh and colleagues [14] proposed the UTAUT2, an extension of the model to the consumer use context and incorporated new constructs, namely hedonic motivation, price value, experience and habit. Longitudinal studies have shown that the UTAUT is able to explain about 70 percent of the variance in the usage intention and about 50 percent of the variance in the actual usage of technology [14]. To date, the entire model or part of the model has been applied to the study of the implementation of different technologies, both in organizational and non-organizational contexts [14].

**Figure 1:**
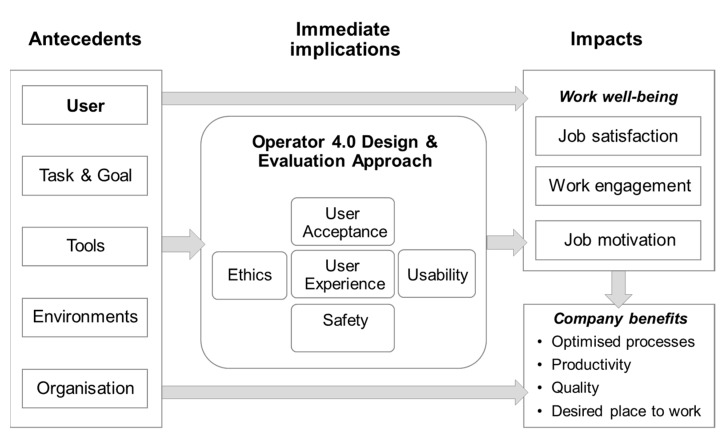
The Worker-Centric Design and Evaluation Framework for Operator 4.0 [21] (p. 267). The figure is licensed under the terms of the Creative Commons Attribution 4.0 International License (http://creativecommons.org/licenses/by/4.0/), which permits use, sharing, adaptation, distribution and reproduction in any medium or format.

More recently, Kaasinen and colleagues [21] proposed a new broader framework that supports design, evaluation and impact assessment of work systems. Contrary to previous models, the Worker-Centric Design and Evaluation Framework for Operator 4.0 (Figure 1) has the advantage of having considered workers’ wellbeing and satisfaction. With this focus, authors particularly referred to Danna and Griffin’s model [22], according to which the wellbeing of workers is influenced by different antecedents (personality traits and work environment) and may impact on individual and organizational outcomes. 

The framework of Kaasinen and colleagues [21] aims to aid the design and evaluation of new systems and tools and to facilitate the assessment of their impact on the wellbeing of workers and organizational outcomes. In this framework, the intervention (e.g., the introduction of a type of new technology) is differentiated by its antecedents and consequences, thus three macro-categories are identified. (1) Antecedents refer to “the original context of use [23] where the intervention—in the form of new tools and new work practices—is implemented” [21] (p. 266). Among antecedents, the framework includes user characteristics, the task and goal involved by the use of the system, other tools, the environment where the user performs his/her activities, and the organizational resources and barriers. (2) Immediate implications encompass workers’ experience with the tool or procedure, in terms of acceptance, usability, safety and ethics. (3) Impacts are the wellbeing of workers and organizational outcomes. Specifically, for the wellbeing of workers, the framework suggests positive outcomes, in other words, job satisfaction, work engagement and job motivation, while company benefits would be optimized processes, productivity, quality, and an improved company image. According to the framework, antecedents may influence the workers’ experience with the new tool, which in turn may affect the workers’ wellbeing and organizational benefits. 

In this study, we used part of the Worker-Centric Design and Evaluation Framework for Operator 4.0 to investigate the antecedents and impacts of technology acceptance. As a wellbeing outcome, we considered work engagement, defined by Schaufeli and colleagues [24] as “a positive, fulfilling, work-related state of mind, which is characterized by vigor, dedication and absorption” (p. 74). According to the motivational process of the job demands–resources model [25], both the job and personal resources may positively affect work engagement, especially in the presence of high job demands. In line with the effort–recovery theory [26], employees’ willingness to dedicate efforts and skills to their work tasks is nurtured by resources provided by the environment. Hence, job resources play an extrinsic motivational role in encouraging compensatory efforts to reduce job demands and accomplish work goals. Moreover, they play an intrinsic motivational role in satisfying individuals’ needs for competence, autonomy and affiliation [27]. Similarly, personal resources, which are defined as “the psychological characteristics or aspects of the self that are generally associated with resiliency and that refer to the ability to control and impact one’s environment successfully” [28] (p. 49), promote the achievement of work goals and encourage personal development. Several cross-sectional and longitudinal studies confirmed the role of both job and personal resources in the motivational process, considering a wide variety of occupational work settings (for an overview, see [28]). 

In the literature, positive associations between work engagement and performance outcomes have been found, such as in-role and extra-role performance, team performance, customer loyalty, quality of service and care [29]; thus, work engagement is broadly considered an advantage for organizations [30,31]. As for immediate implications, we investigated user acceptance and among its antecedents we took into consideration two users’ personal characteristics (resilience and goal orientation) and an organizational resource (opportunities for information and training); both personal and job resources have already been considered in previous studies that investigated the determinants of work engagement [28].

### 1.2. Personal and Oorganizational Antecedents

The Industry 4.0 revolution entails several changes for industries and societies and is contributing to the current perception of the world being a place characterized by Vulnerability, Uncertainty, Complexity and Ambiguity (VUCA) [32]. In order to react to these unexpected changes and to achieve long-term results, organizations and workers need to adapt and to be resilient [33]. Resilience is an individual’s ability to face and overcome frustrating, stressful and adverse situations in adaptive ways, using effective coping strategies. Resilient individuals use positive emotions to deal with negative situations and show a greater capacity for recovery after difficult circumstances [34]. Resilience is considered an important resource in the Industry 4.0 era [35], since “resilient employees are willing, and able, to overcome fears of STARA (Smart Technology, Artificial intelligence, Robotics and Algorithms) by tapping into their emotional strength” [36] (p. 35).

The second personal resource considered in this study is goal orientation, originally defined as a situated orientation for action or a disposition toward developing and demonstrating abilities in the accomplishment of a task [37]. In applied psychology, goal orientation is one of the most studied motivational variables, and has been related to adaptive behaviors in several contexts, including goal setting and performance adaptability [38]. It has been suggested as one of the success factors of adaptable and flexible manufacturing organizations in the Industry 4.0 era [39,40]. 

Among organizational antecedents, opportunities for information and training have been considered. The lack of technical and digital skills and the subsequent need for training is one of the most significant hindrances to the implementation of new innovative technologies [4]. At the same time, the scarcity or inadequacy of information pertaining to the implementation of new systems may contribute to an increase in fears and suspicions that may also obstruct users’ acceptance. Indeed, despite the great advancement in understanding the determinants of technology acceptance, the literature reports examples of IT implementation failures and low availability on behalf of workers to use new systems [17]. 

In order to introduce and implement new systems in an effective way, organizations should develop interventions that can encourage acceptance and compliance among workers [41,42]. A common problem when a transformation occurs in an organization is that people who implement a new system do not consider it to be a major change, while operators who will use it do; as a consequence, the focus on the technological aspect is high, while the focus on the human factor is limited [43]. According to the literature on change management (e.g., [44]), one of the “golden rules” for effective change processes is people’s active participation in the change implementation, also through adequate communication and training [45]. When a new technology is implemented within an organization, the flow of information about the reasons for the change, how it happens and its impacts on workers makes it possible to involve all employees and make them aware. On the other hand, training for workers who will use the new technology is necessary to develop both abilities for its usage and positive attitudes towards the technology itself [11].

**Figure 2:**
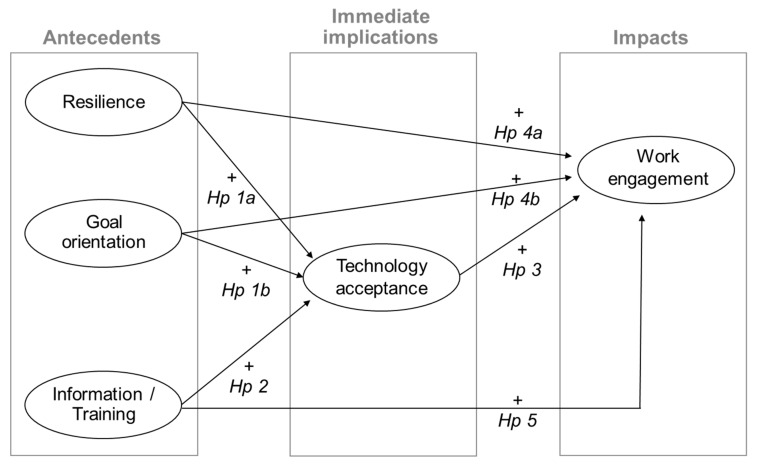
The hypothesized model.

Given these premises, this study intended to investigate how resilience and goal orientation (as user characteristics) and opportunities for information and training received (as organizational resource) are related to technology acceptance, and how the latter is associated with work engagement. Figure 2 shows the hypothesized model that has been investigated simultaneously in white- and blue-collar groups and the study hypotheses are the following.

**Hypothesis 1** **(H1).** *(a) resilience and (b) goal orientation have a positive relationship with technology acceptance in white- and blue-collar workers.*

**Hypothesis 2** **(H2).** *Opportunities for information and training have a positive relationship with technology acceptance in white- and blue-collar workers.*

**Hypothesis 3** **(H3).** *Technology acceptance has a positive relationship with work engagement in white- and blue-collar workers.*

**Hypothesis 4** **(H4).** *(a) resilience and (b) goal orientation have a positive relationship with work engagement in white- and blue-collar workers.*

**Hypothesis 5** **(H5).** *Opportunities for information and training have a positive relationship with work engagement in white- and blue-collar workers.*

## 2. Materials and Methods 

### 2.1. Procedure and Participants

The study was carried out in an Italian manufacturing company and involved two production plants, with similar processes, located in the North of Italy. The company was investing in Industry 4.0 and had recently introduced innovations such as new technologies, automation systems, exoskeletons and robots within the plants. The project was approved by the company Board of Directors and was designed in collaboration with the Health, Safety & Environment Department. Moreover, the project’s aims, methods and tools were shared with the trade unions. The study was carried out in line with the Helsinki Declaration [46] and the Italian data protection regulation. An agreement between the company and the University of Turin’s Department of Psychology was contracted in order to guarantee anonymity and confidentiality in collecting, analysing and publishing data. 

At the two plants, all white-collar workers (257) were invited to fill-in the questionnaire; the participants (220 employees) represented 86% of the whole population of white-collar workers. As for blue-collar employees, a representative sample of the whole population was involved in the research; the final sample (378 participants) included 17% of the whole population (2287 employees). In this study, we have considered only participants whose work activity was recently modified by the introduction of new technologies. 

The participants received a paper questionnaire and an informed consent form, which described the study’s aims, the measures taken to guarantee anonymity, participants’ rights in terms of data protection and their voluntary participation in the study. Questionnaires were administered to the participants on site in small groups during working hours and in the presence of a researcher. Respondents returned their questionnaire in drop-boxes provided by the researcher. 

A total of 598 employees participated in the research. The group of white-collar workers was made up of 220 employees. Among them, 83% were male and 16% were female; the mean age was 43.97 years (SD = 8.67). A total of 68% had a high school diploma; 24% a bachelor’s or master’s degree; and 8% had a lower educational level. The mean level of seniority on the job was 21.33 years (SD = 10.43). The group of blue-collar workers was made up of 378 participants. Among them, 85% were male and 15% were female; the mean age was 42.16 years (SD = 9.60). A total of 50% had a high school diploma; 3% a Bachelor’s or Master’s degree, and 47% had a lower educational level. The mean level of job seniority was 21.33 years (SD = 10.35). 

### 2.2. Measures

Work engagement was measured using eight items of the Oldenburg Burnout Inventory [47]; an example item is “I find my work to be a positive challenge” (Likert scale from 1 = totally disagree to 5 = totally agree). Cronbach’s alpha was 0.80 for the white-collar sample and 0.82 for the blue-collar sample.

Technology acceptance was detected through four items taken from the Subjective Acceptance Questionnaire [48]; an example item is “The use of technology and automation systems increases my professional effectiveness” (Likert scale from 1 = totally disagree to 5 = totally agree). Cronbach’s alpha was 0.75 for the white-collar sample and 0.77 for the blue-collar sample.

Resilience was measured with five items of the Connor and Davidson’s resilience scale [49]; an example item is “I am able to adapt to change” (Likert scale from 1 = Almost always false to 5 = Almost always true). Cronbach’s alpha was 0.75 for the white-collar sample and 0.73 for the blue-collar sample.

Goal orientation was measured through five items of the Italian Motivational Orientation Test [50]; an example item is “I strive to give the best of myself in every situation” (Likert scale from 1 = totally disagree to 5 = totally agree). Cronbach’s alpha was 0.79 for the white-collar sample and 0.75 for the blue-collar sample.

Opportunities for information and training was detected using five ad-hoc items, which investigated how much respondents felt they received adequate opportunities to be informed about their work and to be trained. Table A1 in the Appendix A displays all items; an example is “When I need information, I know where to get it” (Likert scale from 1 = totally disagree to 5 = totally agree). Cronbach’s alpha was 0.84 for the white-collar sample and 0.81 for the blue-collar sample. The Confirmatory Factor Analysis (CFA) confirmed the one-factor structure of the scale: χ^2^ (4) = 9.50; *p* < 0.05; Root Mean Square Error of Approximation (RMSEA) = 0.05 (0.00, 0.08); Comparative Fit Index (CFI) = 0.99; Tucker–Lewis Index (TLI) = 0.99; Standardized Root Mean Square Residual (SRMR) = 0.02. Factor loadings were significant and ranged between 0.50 and 0.84. The covariance between the residuals of item 1 and item 2 (both related to the opportunity to find information) was calculated.

### 2.3. Data Analysis

Descriptive data analysis was performed in the two samples (white-collar and blue-collar) separately. Pearson correlations were calculated to investigate the relationships among variables in each sample. Cronbach’s alpha coefficients were tested to examine the reliability of each measure. The analysis of variance, through *t*-test for independent samples, was considered to investigate differences in the variables’ means between white-collar and blue-collar samples. The above-mentioned analyses were performed through the support of statistics software SPSS 25 (IBM, Armonk, NY, USA). 

Harman’s single-factor test [51] was used to exclude the common method variance issue. CFA results demonstrated that one single factor could not account for the variance in the data (χ^2^ (324, N = 598) = 3230.17, *p* < 0.001, RMSEA = 0.12 (0.12, 0.13), CFI = 0.52, TLI = 0.48, SRMR = 0.10); thus, the common method variance problem was rejected in this study.

The hypothesized model, which compared the two samples, was tested using a multi-group full structural equation model (SEM), performed with the aid of Mplus 7 (Muthén & Muthén, Los Angeles, CA, USA). The method of estimation was Maximum Likelihood (ML). Age and professional seniority were considered in the model as control variables. The item parcelling technique [52] was adopted for all variables in the measurement model; parcels were created on the basis of the calculated mean between different items referring to the same construct. 

The model was assessed by the following goodness-of-fit criteria [53]: the χ^2^ goodness-of-fit statistic (non-significant values indicate that the hypothesized model fits the data); the RMSEA (values smaller than 0.05 indicate a good fit, values smaller than 0.08 indicate an acceptable fit and values greater than one should lead to model rejection); the CFI and the TLI (CFI and TLI values greater than 0.90 indicate an acceptable fit, and values greater than 0.95 indicate a good fit); the SRMR (values higher than 0.08 indicate a poor fit to the empirical data, values lower than 0.05 indicate an excellent fit); and AIC (smaller values indicate better models). The significance of the indirect effects was tested through a bootstrapping procedure which extracted 2000 new samples from the original one in order to calculate the direct and indirect parameters of the model [54].

## 3. Results

Means, standard deviations, and correlations between the study variables are presented in Table 1 for the whole sample and Table 2 for the two groups separately. In the three cases, results showed a significant and positive correlation between work engagement and the four considered variables, namely technology acceptance, resilience, goal orientation and opportunities for information and training. Technology acceptance positively correlated with resilience, goal orientation and opportunities for information and training in the whole sample and in the two groups; moreover, it showed a negative correlation with both age and professional seniority in the whole sample and in the blue-collar workers group, while these correlations were not significant in the white-collar workers group.

The analysis of variance between the white-collar and blue-collar groups showed a significant difference in the technology acceptance variable, with a higher level for white-collar (*M* = 3.95) compared with blue-collar (*M* = 3.81) (*t* (562) = 6.45, *p* < 0.001) workers. Moreover, goal orientation was significantly higher for white-collar (*M* = 4.21) than for blue-collar (*M* = 4.09) (*t* (536) = 2.24, *p* = 0.026) workers.

**Figure 3:**
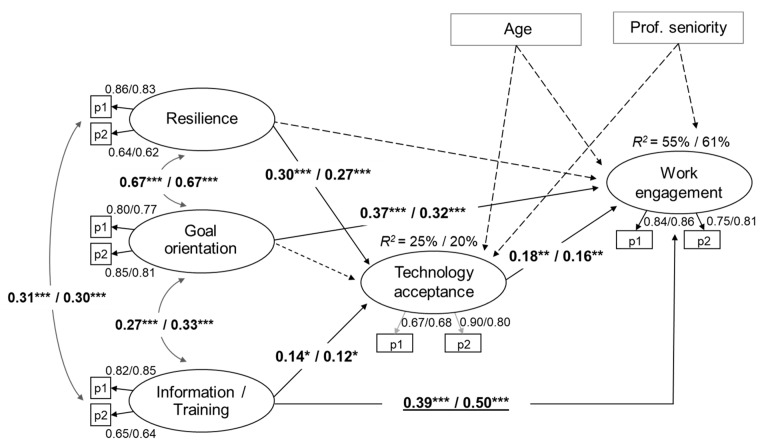
The final model M_3_ (standardized path coefficients; white-collar data/blue-collar data). Discontinuous lines indicate non-significant relationships. Underlined data are statistically different between white-collar and blue-collar. *** *p* < 0.001; ** *p* < 0.01; * *p* < 0.05. *p* < 0.001 for all factor loadings.

Table 3 presents the results of alternative models. M_1_ represents the hypothesized model, investigated through a multi-group full SEM with all parameters constrained to be equal across groups; the model fitted to the data well: χ^2^ (103) = 255.99, *p* < 0.001, CFI = 0.93, TLI = 0.91, RMSEA = 0.07 (90% CI 0.05, 0.08), SRMR = 0.07. M_2_, an alternative model without the mediation of technology acceptance, showed a significantly worse fit to the data than M_1_. After examination of the modification indices of M_1_, it appeared that, when releasing the equality constraint of the relationship between opportunities for information and training and work engagement, the fit model significantly improved. The final model (M_3_) with the mediation of technology acceptance and one free structural parameter had the best fit to the data: χ^2^ (102) = 250.67, *p* < 0.001, CFI = 0.93, TLI = 0.91, RMSEA = 0.07 (90% CI 0.05, 0.08), SRMR = 0.07. In the final model, which is shown in Figure 3, the latent variables were well-defined with factor loadings of the observed variables ranging between 0.62 and 0.90. In the model, both resilience and opportunities for information and training showed a positive relationship with technology acceptance in both groups. In turn, technology acceptance was positively related to work engagement in both groups. Moreover, both goal orientation and opportunities for information and training had a positive association with work engagement; the relationship between opportunities for information and training and work engagement was significantly higher for blue-collar workers than white-collar. All three independent variables positively correlated with each other in both groups. The model explained about 25% and 20% of the variation in technology acceptance respectively for white-collar and blue-collar workers, and 55% and 61% of the variation in work engagement respectively for white-collar and blue-collar workers.

Table 4 shows the statistically significant indirect effects evaluated through the bootstrapping procedure. According to results, technology acceptance fully mediated the relationship between resilience and work engagement in both groups. Moreover, the relationship between opportunities for information and training and work engagement was partially mediated by technology acceptance in both groups. 

## 4. Discussion

The study contributes to the literature on technology acceptance in different ways: (1) it investigated its antecedents considering personal and organizational resources; (2) it is one of the first that considered the relationship of technology acceptance with a dimension of workers’ wellbeing, namely work engagement; and (3) it is one of the few studies in work and organizational psychology literature that involved blue-collar workers and compared them with white-collar colleagues. Moreover, findings supported the use of the Worker-Centric Design and Evaluation Framework for Operator 4.0 [21] in order to investigate the implementation of new technologies within factories focusing on users’ characteristics and their wellbeing.

Among the personal resources, only resilience showed a positive relationship with technology acceptance in both groups; therefore, Hypothesis 1a was confirmed, while Hypothesis 1b was rejected. The implementation of new technologies and/or new tools means an important change for workers and, in some cases, entails difficulties, especially at the beginning. Thus, the capability to face adverse situations and overcome them in an adaptive way, typical of resilient people [34], is one of the critical personal characteristics for workers in Industry 4.0, since it may foster the acceptance of new technologies. Moreover, this characteristic seems to be more important than workers being oriented towards their own goals when coping with technology changes introduced into their work.

Opportunities for information and training—the organizational resource considered in this study—showed a positive association with technology acceptance in both groups, confirming Hypothesis 2. This result reinforces the importance of considering the implementation of new tools and systems in organizations as a change process, which requires adequate communication and training to involve workers in a positive and effective way [45]. Having the opportunity to be informed and trained about the introduction of new technologies, whatever they are, allows employers to deal with workers’ doubts and fears and to involve them. In this way, workers do not believe that the change is being done to them, they feel part of it. In this study, both resilience and opportunities for information and training can be defined as perceived resources [18] or facilitating conditions [19] able to support technology acceptance. 

The positive relationship between technology acceptance and work engagement was also confirmed in white- and blue-collar groups (Hypothesis 3). As far as we know, few studies have considered workers’ wellbeing as a consequence of technology acceptance, despite the great attention paid to the concept of human-centricity in the factories of the future [55]. As we already know, having a positive attitude towards the new technologies that workers are required to use in their activities facilitates their effective usage [14]. In addition to this, our findings support the idea that a positive disposition towards the tools may encourage work engagement. In other words, technology acceptance is a resource able to foster the motivational process, making workers more energetic, willing to invest effort in their activities and persistent in the face of problems or difficulties [24]. According to the Conservation of Resources Theory [56], engaged workers are able to generate and reinforce their resources, creating a positive spiral [57] that could also include technology acceptance. Future longitudinal studies are needed to investigate this assumption; however, it represents an important suggestion for organizations in Industry 4.0 that will ask their workers to face several changes. 

With regards to the relationship between personal resources and work engagement, only goal orientation showed a positive association, confirming Hypothesis 4b. This result is supported by the job demands–resources model [25], which assumed that personal resources increase work engagement. According to the model, “the higher an individual’s personal resources, the more positive the person’s self-regard and the more goal self-concordance is expected to be experienced [58]. Individuals with goal self-concordance are intrinsically motivated to pursue their goals and as a result they trigger higher performance and satisfaction” [59] (p. 13). Contrary to our expectations, this is not applicable for resilience in our study; indeed, Hypothesis 4a was rejected. However, resilience showed a positive relationship with work engagement fully mediated by technology acceptance. It seems that a resource like resilience plays a crucial role particularly in stressful situations, such as the implementation of new technologies; in this case, resilience promotes work engagement, fostering a positive attitude towards the change. 

Finally, the positive relationship between opportunities for information and training and work engagement (Hypothesis 5) was confirmed in both groups. This result is in line with previous studies that suggested opportunities for development and information as two of the core job resources involved in the motivational process described by the job demands–resources model [28,59]. In our study, the relationship between opportunities for information and training and work engagement was also partially mediated by technology acceptance, which is line with the idea that job conditions that support professional development, such as information and training, allow employees to face continuous changes and to feel involved in achieving their work goals [60]. Furthermore, results showed that the association between opportunities for information and training and work engagement was significantly higher for blue- compared with white-collar workers. At the same time, contrary to previous studies [61], we did not find different levels of work engagement in the two groups. In summary, our result confirms that some differences exist between the two populations, differences that also involved motivational dynamics and their antecedents, but more research is needed to better investigate and understand it. 

### Limitations

A first limitation of this study is its cross-sectional design, as longitudinal or diary studies would be necessary to test causal relationships across time. In addition, this kind of method would be useful to monitor the effects of training and other interventions over time. A second limitation is that only self-reported data has been considered, supporting the risk of common method-bias [51]. Despite the difficulty in collecting other types of data within organizations, future studies on these topics should also include other-reported (e.g., supervisors and colleagues) and/or objective data (e.g., training hours).

As for the model that the study investigated, a limited set of variables has been considered, while other personal resources, such as self-efficacy and flexibility, may play an important role. Moreover, it would be notably important to investigate the role of supervisors and their leadership style with multilevel studies. Finally, the study involved employees who use different tools in their work activities; therefore, the acceptance construct was not related to a common technology. Nevertheless, the study permitted us to consider a large representative sample of white- and blue-collar workers employed in the same company, providing useful suggestions, especially for HR practice, which are discussed below.

## 5. Conclusions

In summary, this study contributed to the literature showing the role of certain personal and organizational resources as antecedents of technology acceptance. Moreover, it highlighted the connection between technology acceptance and work engagement, clarifying how important acceptance is not only in fostering effective usage of the new technology by workers, but also in preventing and encouraging their wellbeing. 

From a practical point of view, these findings suggest useful and primary implications for organizations, HR managers and supervisors. First of all, in accordance with change management models, when a new technology is introduced, adequate opportunities for information and training should be offered to all workers. These strategic interventions should encompass three levels. The first level includes a communication and information program for the company population at large, in order to anticipate and prepare for the change and promote collective awareness and involvement. The second level regards training for all workers to improve their knowledge, technical and soft skills and to embrace their doubts and fears; this type of training should not be directly relevant to workers’ jobs, but is useful for increasing the variety of skills [62]. Finally, the third and more specific level involves developing effective users of the new tools with specific training courses aimed at teaching how to use the technology. In training programs, soft skills and personal characteristics deserve attention as much as technical skills, since they represent protective factors in changing situations. All these interventions may foster technology acceptance within the company and provide knowledge about Industry 4.0 at all levels. Furthermore, given the pervasiveness of technology in every context, it can be expected that organizational training can have spillover effects in the rest of life, providing the chance to develop skills that people can use in different life domains.

When introducing new interventions or changes, blue-collar workers require particular attention by management. Their profession is characterized by repetition, highly standardized processes and poor communication. Nevertheless, an adequate level of autonomy and a supportive climate should be guaranteed through commensurate opportunities to be informed and trained [63]. Moreover, a crucial role in change process is played by leaders and supervisors; they can be supported in dealing with the transformations and the challenging requirements of the Industry 4.0 era [1] through specific interventions, which include classroom training, mentoring and individual or team coaching.

In conclusion, the ongoing transformations imply a progressive and continuous change of processes and technologies, which in turn requires new skills and new working assignments. In this scenario, employees play a fundamental role; involving them in the development and implementation of new systems is crucial, for example, by asking them to propose suggestions to make improvements to their working environment (as encouraged by some lean production models, e.g., the World Class Manufacturing model [64]). Finally, if management actually adopts people’s points of view, turning them into technologies and skills development, using a co-construction approach, employees will feel motivated and engaged.

## Figures and Tables

**Table 1:** Means, standard deviations, Cronbach’s alphas and correlations among study variables.

Variables	1	2	3	4	5	6	7
1. Work engagement	0.82						
2. Technology acceptance	0.32 **	0.76					
3. Resilience	0.37 **	0.29 **	0.73				
4. Goal orientation	0.44 **	0.31 **	0.50 **	0.78			
5. Opp. for information and training	0.50 **	0.23 **	0.25 **	0.24 **	0.81		
6. Age	−0.03	−0.16 **	−0.10 *	−0.22 **	0.02	-	
7. Professional seniority	−0.03	−0.15 **	−0.12 **	−0.17 **	0.02	0.69 **	-
M	3.60	3.86	3.83	4.14	3.34	42.82	21.33
SD	0.80	0.82	0.69	0.69	0.83	9.31	10.37

Note: Cronbach’s *α* on the diagonal. ** *p* < 0.01; * *p* < 0.05.

**Table 2:** Means, standard deviations, Cronbach’s alphas and correlations among study variables for white-collar and blue-collar workers.

Variables	1	2	3	4	5	6	7
1. Work engagement	0.78/0.82	0.29 **	0.38 **	0.45 **	0.53 **	−0.02	−0.05
2. Technology acceptance	0.39 **	0.75/0.77	0.28 **	0.31 **	0.23 **	−0.19 **	−0.18 **
3. Resilience	0.36 **	0.32 **	0.74/0.73	0.50 **	0.24 **	−0.08	−0.13 *
4. Goal orientation	0.42 **	0.29 **	0.51 **	0.80/0.77	0.26 **	−0.21 **	−0.18 **
5. Opp. for information and training	0.42 **	0.21 **	0.25 **	0.20 **	0.84/0.79	0.03	0.05
6. Age	−0.05	−0.10	−0.13	−0.25 **	−0.01	-	0.68 **
7. Professional seniority	−0.05	−0.12	−0.09	−0.19 **	−0.05	0.71 **	-
White-collar							
M	3.84	3.95	3.84	4.21	3.42	43.97	21.33
SD	0.63	0.67	0.64	0.59	0.77	8.67	10.43
Blue-collar							
M	3.45	3.81	3.82	4.09	3.29	42.16	21.33
SD	0.85	0.89	0.71	0.74	0.86	9.60	10.35

Note: Correlations for the white-collar group below the diagonal; correlations for the blue-collar group above the diagonal. Cronbach’s α for white-collar/blue-collar groups on the diagonal. ** *p* < 0.01; * *p* < 0.05.

**Table 3:** Results of alternative SEMs.

Models	χ^2^	*df*	*p*	CFI	TLI	RMSEA	SRMR	AIC	Comparison	Δχ^2^	*p*
**M_1_.**	255.99	103	<0.001	0.93	0.91	0.07 (0.05, 0.08)	0.07	12,997.21			
**M_2_.**	262.53	102	<0.001	0.92	0.90	0.07 (0.06, 0.08)	0.08	13,005.76	M_2_ − M_1_	6.54	0.011
**M_3_.**	250.67	102	<0.001	0.93	0.91	0.07 (0.05, 0.08)	0.07	12,993.90	M_1_ − M_2_	5.32	0.021

Note: M_1_ is the hypothesized constrained model with technology acceptance as mediator. M_2_ is the direct effects model without mediation of technology acceptance. M_3_ is the hypothesized constrained model with technology acceptance as a mediator and the parameter opportunities for information and training (Inf/Train) → work engagement (WE) released. Comparative Fit Index (CFI). Tucker–Lewis Index (TLI). Root Mean Square Error of Approximation (RMSEA). Standardized Root Mean Square Residual (SRMR). Akaike’s Information Criterion (AIC).

**Table 4:** Indirect effects using bootstrapping (2000 replications).

Indirect Effects—White-Collar	Est.	SE	*p*	CI 95%
Res → Tech → WE	0.05	0.02	0.020	(0.01, 0.12)
Inf/Train → Tech → WE	0.04	0.01	0.038	(0.01, 0.06)
**Indirect Effects—Blue-Collar**	**Est.**	**SE**	***p***	**CI 95%**
Res → Tech → WE	0.05	0.02	0.019	(0.01, 0.10)
Inf/Train → Tech → WE	0.03	0.01	0.039	(0.01, 0.05)

Note: All parameter estimates are presented as standardized coefficients. Estimates (Est.). Standard Error (SE). Confidence interval (CI). Resilience (Res). Technology acceptance (Tech). Opportunities for information and training (Inf/Train). Work engagement (WE).

## Appendix A

**Table A1:** Opportunities for information and training measures.

Thinking about Your Working Situation, How Much Do You Agree with The Following Statements?
1. It is easy to get the information I neeSd
2. When I need information, I know where to get it
3. Professional update opportunities are adequate
4. Provided training is adequate
5. I can learn new things and professionally grow

Note: Likert scale from 1 = totally disagree to 5 = totally agree.
